# Genial Tubercle Avulsion in a Traumatic Mandibular Injury

**DOI:** 10.7759/cureus.104752

**Published:** 2026-03-06

**Authors:** Panagiotis Giasimakopoulos, Dimitris Tatsis, Anestis Chrysostomidis, Asterios Antoniou, Konstantinos Paraskevopoulos

**Affiliations:** 1 Oral and Maxillofacial Surgery, General Hospital of Thessaloniki “George Papanikolaou”, Thessaloniki, GRC; 2 Oral and Maxillofacial Surgery, Aristotle University of Thessaloniki, Thessaloniki, GRC

**Keywords:** facial trauma, genial tubercle avulsion, mandibular fracture, maxillofacial surgery, motorcycle accident, orif, sublingual emphysema, symphyseal fracture

## Abstract

Mandibular fractures represent a common consequence of high-energy facial trauma; however, avulsive fractures of the genial tubercle are exceedingly rare and sparsely documented in the literature. These injuries may be easily overlooked, particularly in polytrauma settings, despite their potential impact on airway function and mandibular stability. This report describes a unique case of combined midfacial and mandibular trauma, including an uncommon 2-cm avulsion fracture of the genial tubercle, following a motorcycle accident. A 22-year-old male presented to the emergency department after a motorcycle collision at approximately 50 km/hour. Initial evaluation revealed abrasions, intraoral lacerations, and mild left periorbital swelling. CT imaging demonstrated fractures of the anterior wall of the left maxillary sinus and the left orbital floor, a mandibular symphyseal fracture extending from #33 to #43, and a rare avulsive fracture of the genial tubercle associated with sublingual emphysema. Ophthalmologic, otolaryngologic, and neurosurgical examinations revealed no functional deficits or intracranial pathology. The patient received tetanus prophylaxis, systemic antibiotics, corticosteroids, and topical ophthalmic therapy. Surgical management included open reduction and internal fixation of the symphyseal fracture with two 2.0-mm four-hole plates under general anesthesia, preceded by stabilization with an Essig-type arch bar. Postoperative recovery was uneventful, and follow-up examinations confirmed satisfactory occlusion, stable fixation, and progressive healing. The Essig splint was removed six weeks after surgery. Long-term follow-up at three years confirmed radiographic stability of the genial tubercle fragment without secondary displacement or resorption, and the patient remained asymptomatic. This case highlights an exceptionally rare traumatic injury pattern involving an avulsive fracture of the genial tubercle. Early identification, appropriate imaging, and timely interdisciplinary assessment are essential for optimal management. This report adds to the limited literature on genial tubercle fractures and underscores the importance of maintaining clinical suspicion in anterior mandibular trauma.

## Introduction

Genial tubercle fractures represent an exceptionally rare subtype of anterior mandibular trauma. In contrast to common mandibular fracture sites such as the condyle, angle, or body, injuries involving avulsion or fragmentation of the genial tubercle occur infrequently and are documented primarily in isolated case reports and small series [1-5]. Their rarity is attributed to the deep midline location of the genial tubercle, which is shielded internally by the musculature of the floor of the mouth and externally by the dense cortical bone of the mandibular symphysis. When a fracture does occur, it often results from a combination of direct impact to the anterior mandible and abrupt traction exerted by the suprahyoid musculature, particularly the genioglossus and geniohyoid muscles, which attach at the genial tubercle and may displace fractured fragments posteriorly or inferiorly [1-4].

Spontaneous fractures of the genial tubercle have also been reported, typically associated with underlying bone atrophy, denture instability, or chronic muscular stress [4-13]. However, traumatic fractures, especially those associated with high-energy mechanisms such as motorcycle accidents, remain uncommon [10,14]. Reported clinical presentations range from mild anterior floor-of-mouth discomfort to significant pain, sublingual hematoma, dysphagia, and limited tongue mobility [14]. Because symptoms may be subtle or overshadowed by concomitant midfacial injuries, genial tubercle fractures are frequently underdiagnosed during the acute trauma evaluation [10].

Imaging plays a critical role in identifying these fractures. Conventional radiographs may fail to reveal small avulsive fragments, whereas CT provides accurate delineation of bony pathology, fragment displacement, and associated soft-tissue emphysema [10]. In many cases, genial tubercle fractures coexist with other injuries such as symphyseal fractures, dentoalveolar trauma, or midfacial fractures, features that may alter treatment planning [11]. Early recognition is essential, particularly in the context of trauma, as missed or untreated fractures may result in ongoing pain, speech alteration, dysphagia, or, rarely, airway instability [14].

Management strategies vary depending on fracture morphology, degree of displacement, and presence of associated mandibular fractures. Minor, non-displaced fractures may be treated conservatively, whereas displaced or symptomatic fractures, especially those accompanied by symphyseal instability, often require open reduction and internal fixation (ORIF) to restore mandibular continuity and muscular function [10,14]. Despite general treatment principles, the limited number of published cases makes it challenging to standardize management guidelines.

The present case involves a 22-year-old male who sustained multiple facial fractures following motorcycle trauma, including injuries of the anterior maxillary sinus wall, orbital floor, mandibular symphysis, and a 2-cm avulsive fracture of the genial tubercle with associated sublingual emphysema and hematoma. This unusual injury pattern depicts the diagnostic complexity of anterior mandibular trauma and illustrates key considerations in the surgical management of genial tubercle fractures.

## Case presentation

A 22-year-old male with an unremarkable medical and surgical history presented to the emergency department following a motorcycle collision. The accident occurred at an estimated speed of 50 km/hour. Τhe patient was wearing an open-face helmet and reported a direct impact to the face upon falling forward. He remained conscious throughout the event and did not report vomiting, amnesia, or other neurological symptoms at the scene. Initial evaluation at a secondary-tier hospital documented a Glasgow Coma Scale score of 15/15. The patient was clinically stabilized and subsequently transferred to the tertiary hospital for further management.

On arrival, the patient was alert, hemodynamically stable, and in no acute distress. Extraoral examination revealed mild swelling and ecchymosis over the left infraorbital region and lower eyelid. Several abrasions were noted along the chin and left malar area. Intraoral inspection demonstrated edema and hematoma of the floor of the mouth, as well as mucosal lacerations along the mandibular anterior vestibule. Palpation elicited tenderness across the symphyseal region, although no mobility was initially appreciable due to soft-tissue edema. The patient’s occlusion appeared altered, with a mild anterior open bite. Although sublingual emphysema was identified on imaging, neither radiographic airway stenosis nor clinical signs of respiratory distress were observed during the acute hospitalization or follow-up period.

A contrast-enhanced CT scan of the face and mandible revealed multiple fractures. These included a fracture of the anterior wall of the left maxillary sinus, a nondisplaced fracture of the left orbital floor, and a symphyseal mandibular fracture extending horizontally across the region corresponding to teeth #33 to #43. Notably, imaging demonstrated a 2-cm avulsive fracture of the genial tubercle, with a small bone fragment displaced and associated with emphysema of the tongue base and floor of the mouth. No intracranial injuries, cervical spine trauma, or thoracolumbar spinal abnormalities were identified as per Advanced Trauma Life Support guidelines.

The ophthalmologic evaluation demonstrated 10/10 vision bilaterally, full painless extraocular movements, and normal anterior and posterior segment findings, with no diplopia or ocular trauma; topical tobramycin-dexamethasone was initiated. Otolaryngology noted marked edema and hematoma of the oral floor but an unremarkable anterior rhinoscopic examination.

**Figure 1:**
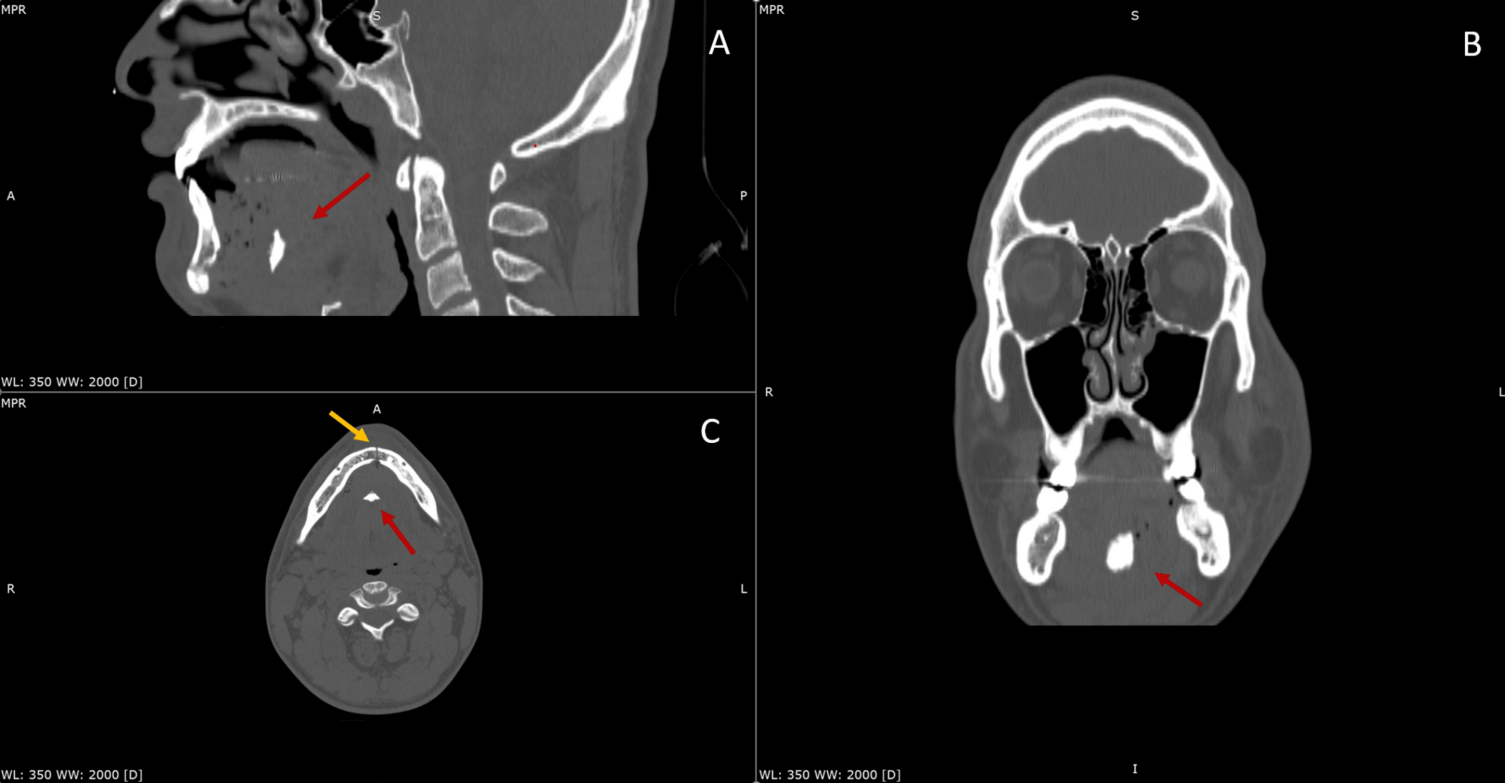
Multiplanar CT imaging of the mandible demonstrating the avulsive genial tubercle fracture and associated anterior mandibular injury. (A) Sagittal CT section showing the avulsed genial tubercle fragment (red arrow) displaced posteriorly toward the floor of the mouth. (B) Coronal CT section confirming the inferior and posterior displacement of the genial tubercle fragment (red arrow) in relation to the mandibular symphysis. (C) Axial CT section demonstrating the avulsive genial tubercle fragment (red arrow) and the midline mandibular fracture at the symphyseal region (yellow arrow), with associated sublingual emphysema.

Given the mandibular instability and the presence of combined symphyseal and genial tubercle fractures, the decision was made to proceed with surgical management. Surgery was performed within 24 hours of the injury. Following nasotracheal intubation, an Essig-type arch bar was placed to achieve temporary stabilization and restore the patient’s occlusion via maxillomandibular fixation. The genial tubercle fracture and the avulsed bony fragment were identified deep to the genioglossus muscle insertion. The symphyseal fracture was reduced anatomically and stabilized using two 2.0-mm titanium plates with four screws each (9-11 mm in length). Stable occlusion was confirmed intraoperatively before flap closure and suturing. The genial fragment was left in situ due to its small size and non-displaced position. The patient was extubated uneventfully and transferred to the surgical ward for postoperative observation until discharge (Figure 1).

The patient was discharged with instructions for a soft diet, oral hygiene, and close follow-up. At the 14th postoperative day, surgical sites demonstrated satisfactory healing, and occlusion remained stable. The Essig splint was maintained to ensure continued stabilization. On the 28th postoperative day, the Essig splint was removed without complication. At that time, the patient exhibited normal tongue mobility, full mandibular function, and no residual symptoms related to the genial tubercle fracture.

This constellation of findings, particularly the avulsive genial tubercle fracture, is exceptionally rare and underscores the importance of thorough radiologic evaluation in cases of anterior mandibular trauma.

**Figure 2:**
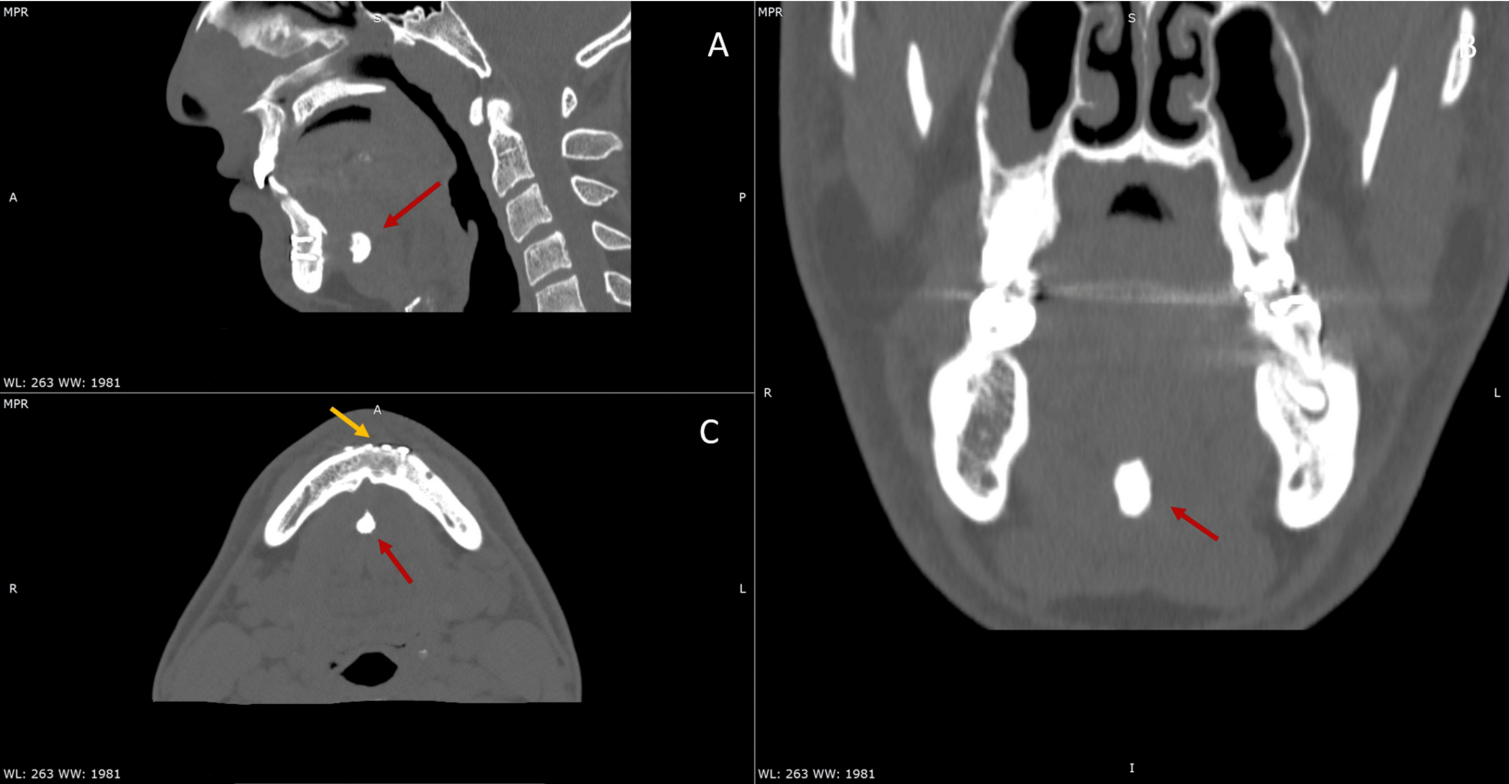
Three-year follow-up multiplanar reconstructed CT images demonstrating radiographic stability of the previously documented injury. (A) Sagittal view showing persistence of the avulsed genial tubercle fragment within the floor of the mouth (red arrow), without significant migration or resorption. (B) Coronal view confirming stable healing of the associated midfacial fractures, with preserved orbital anatomy, genial tubercle fragment (red arrow) in relation to the mandibular symphysis. (C) Axial view illustrating a healed midline mandibular symphyseal fracture (yellow arrow) and unchanged position of the avulsed genial tubercle fragment in the sublingual space (red arrow).

At the three-year follow-up, the patient was successfully re-evaluated clinically and radiographically. A follow-up CT scan demonstrated no significant change in the position or morphology of the avulsed genial tubercle fragment, with stable bony configuration and no evidence of secondary displacement, resorption, or pathological remodeling. Clinically, the patient remained asymptomatic, with preserved mandibular function, normal tongue mobility, and no airway-related complaints (Figure 2).

## Discussion

Fractures of the genial tubercle are rare clinical entities and are significantly less common than fractures involving other regions of the mandible, such as the condyle, angle, or parasymphysis. Most of the available literature on genial tubercle fractures consists of isolated case reports and small series, highlighting their infrequency and the resulting paucity of standardized diagnostic and therapeutic guidelines [1-5]. The rarity of these fractures is attributed to the protected location of the genial tubercle on the lingual aspect of the mandibular symphysis, surrounded by thick cortical bone and reinforced by the muscular attachments of the genioglossus and geniohyoid muscles. When injury does occur, it is typically the result of either a significant anterior mandibular impact or spontaneous failure associated with chronic stress, atrophy, or denture instability, the latter being reported more commonly in elderly and edentulous patients [4-10]. In our case, the patient was fully dentate, which makes it rather uncommon for this type of fracture.

In the present case, the patient sustained a high-energy motorcycle trauma, a mechanism consistent with mandibular symphyseal fractures but uncommonly associated with avulsive genial tubercle fractures. Traumatic avulsion of the genial tubercle is believed to result from a combination of direct impact and strong traction forces exerted by the suprahyoid musculature at the moment of injury [1-4]. The genioglossus and geniohyoid muscles, which attach directly to the genial tubercle, may generate significant tensile forces during abrupt deceleration or direct blows, thereby contributing to displacement of the fractured segment. This mechanism has been previously suggested in reports describing anterior mandibular trauma associated with dysphagia, tongue mobility restriction, or airway compromise resulting from posterior displacement of the fragment [10,14]. In our case, the presence of sublingual emphysema and hematoma in the floor of the mouth supports the likelihood of localized soft-tissue disruption accompanying the avulsive fracture. We suspect that the patient, during the high-impact fall from the motorcycle, had a direct blow to the genial region, which resulted in this avulsive fracture.

Clinical presentation of genial tubercle fractures is highly variable. Some patients exhibit minimal symptoms, while others may demonstrate floor-of-mouth pain, dysphagia, speech alteration, or mechanical restriction of tongue movements [10-12]. Edema of the floor of the mouth is a known associated finding in traumatic genial tubercle fractures and may contribute to discomfort or dysphagia [14]. Airway obstruction via the exerted pulling force of the genioglossus muscle, though rare, has been documented when displacement of the genial fragment encroaches upon the oropharyngeal space [14,15]. In the current case, despite the presence of associated fractures of the maxillary sinus wall and orbital floor, the patient did not develop airway compromise or significant functional limitation. This underlines the fact that genial tubercle fractures may be clinically subtle and can easily be overlooked if comprehensive imaging is not performed.

Radiographic evaluation is essential for accurate diagnosis. Conventional radiographs often fail to detect genial tubercle fractures due to the superimposition of soft tissues and the small size of the avulsed fragments [1,10]. CT, particularly multidetector CT with soft-tissue evaluation, remains the most reliable modality to identify the presence, displacement, and extent of the fracture, as well as associated soft-tissue emphysema or concurrent mandibular fractures [10]. In this case, CT clearly demonstrated the avulsive fracture along with additional maxillofacial injuries, guiding the decision-making process for surgical intervention [15].

Management strategies for genial tubercle fractures depend on fracture morphology, displacement, associated injuries, and functional symptoms. Stable, nondisplaced fractures may be treated conservatively, while displaced fractures, especially those associated with symphyseal instability, generally require ORIF [10-12,14]. The symphyseal fracture in this patient necessitated ORIF with load-sharing titanium plates to restore mandibular continuity and occlusion. The genial tubercle fragment, however, was left untreated surgically due to its small size and lack of significant displacement, a management approach supported by previous reports indicating that fixation is not always required for minimally displaced fragments that do not threaten airway patency or muscular function [3,5]. This selective approach minimized surgical morbidity while maintaining functional stability.

The postoperative course was uneventful, consistent with outcomes reported in similar cases where patients regained full mandibular function, tongue mobility, and stable occlusion following appropriate management [1,10]. The absence of postoperative complications such as infection, nonunion, or persistent dysphagia further supports the appropriateness of the chosen treatment strategy.

This case adds to the limited but growing body of literature describing traumatic genial tubercle fractures in young patients. Most existing reports describe spontaneous fractures in older or edentulous individuals [4-10], making this trauma-associated case particularly noteworthy. Additionally, the coexistence of maxillary sinus fractures emphasizes the need for thorough interdisciplinary evaluation in facial trauma cases.

The present case also reinforces the importance of CT imaging in the evaluation of anterior mandibular trauma, given that genial tubercle fractures may otherwise be missed clinically. Furthermore, it highlights the biomechanical significance of the genial region, which plays a crucial role in tongue posture, airway maintenance, and mandibular stability. Although the genial tubercle fragment did not require fixation in this case, clinicians must remain alert to potential complications in similar injuries.

Overall, this case contributes valuable insight into the mechanism, diagnosis, and management of traumatic genial tubercle fractures and underscores the significance of maintaining a high index of suspicion when evaluating anterior mandibular trauma, particularly in the context of high-energy injuries.

Importantly, long-term radiographic follow-up at three years in the present case demonstrated radiographic stability of the genial tubercle fragment without progression, supporting conservative management of minimally displaced avulsive fragments when airway patency and function are preserved.

## Conclusions

Genial tubercle fractures represent an uncommon and underrecognized component of anterior mandibular trauma. Their clinical presentation can be subtle, and diagnosis often requires high-resolution imaging, as conventional radiographs may fail to detect small avulsive fragments. This case illustrates how high-energy trauma may produce a complex injury pattern involving both the mandibular symphysis and the genial tubercle. Prompt identification, interdisciplinary evaluation, and timely surgical management contributed to a favorable postoperative course and full functional recovery. Long-term follow-up in the present case further confirmed radiographic stability and sustained functional outcome. Given the scarcity of such cases in the literature, this report provides valuable insight into the biomechanics, diagnostic challenges, and management considerations of traumatic genial tubercle fractures. Continued documentation of similar cases will help refine clinical awareness and guide future therapeutic strategies.
